# Combination chemotherapy with taxane and platinum in patients with salivary gland carcinoma: a retrospective study of docetaxel plus cisplatin and paclitaxel plus carboplatin

**DOI:** 10.3389/fonc.2023.1185198

**Published:** 2023-06-15

**Authors:** Ryutaro Onaga, Tomohiro Enokida, Kazue Ito, Yuri Ueda, Susumu Okano, Takao Fujisawa, Akihisa Wada, Masanobu Sato, Hideki Tanaka, Naohiro Takeshita, Nobukazu Tanaka, Yuta Hoshi, Makoto Tahara

**Affiliations:** ^1^ Department of Head and Neck Medical Oncology, National Cancer Center Hospital East, Kashiwa, Japan; ^2^ Department of Head and Neck Oncology, Miyagi Cancer Center, Natori, Japan; ^3^ Department of Otorhinolaryngology-Head and Neck Surgery, Tokyo Medical University, Shinjuku, Japan; ^4^ Department of Otorhinolaryngology, Nagoya University Graduate School of Medicine, Nagoya, Japan

**Keywords:** salivary gland carcinoma, cytotoxic chemotherapy, docetaxel, cisplatin, adenoid cystic carcinoma

## Abstract

**Background:**

Despite advances in precision medicine, most patients with recurrent or metastatic salivary gland carcinoma still need conventional chemotherapies, such as the combination of taxane and platinum. However, evidence for these standardized regimens is limited.

**Methods:**

We retrospectively reviewed patients with salivary gland carcinoma treated with a taxane and platinum, which contained docetaxel at a dose of 60 mg/m2 plus cisplatin at a dose of 70 mg/m2 on day 1, or paclitaxel at a dose of 100 mg/m2 plus carboplatin at a dose of area under the plasma concentration-time curve = 2.5 on days 1 and 8 (both on 21-day cycles), between January 2000 and September 2021.

**Result:**

Forty patients with ten adenoid cystic carcinomas and thirty other pathologies were identified. Of these, 29 patients were treated with docetaxel plus cisplatin and 11 with paclitaxel plus carboplatin. For the total population, the objective response rate (ORR) and median progression-free survival (mPFS) were 37.5% and 5.4 months (95% confidence interval: 3.6–7.4 months), respectively. On subgroup analysis, docetaxel plus cisplatin provided favorable efficacy compared with paclitaxel plus carboplatin (ORR: 46.5% *vs.* 20.0%, mPFS: 7.2 *vs.* 2.8 months), and the findings were well retained in patients with adenoid cystic carcinoma (ORR: 60.0% *vs.* 0%, mPFS: 17.7 *vs.* 2.8 months). Grade 3/4 neutropenia was relatively frequent in the docetaxel plus cisplatin (59% *vs.*27%), although febrile neutropenia was uncommon (3%) in the cohort. No treatment-related death was seen in any case.

**Conclusion:**

The combination of taxane and platinum is generally effective and well-tolerated for recurrent or metastatic salivary gland carcinoma. In contrast, paclitaxel plus carboplatin appears unfavorable in terms of efficacy in certain patients, such as those with adenoid cystic carcinoma.

## Introduction

Salivary gland carcinoma (SGC) is a rare malignant tumor which accounts for fewer than 5% of head and neck cancers (1). The disease is classified into over twenty histological types (2), each of which has a distinctive clinical course. In general, surgery and radiotherapy are performed for patients with local disease, whereas systemic therapy is used for those in local treatment is unsuitable, such as subjects with distant metastatic disease (3). However, because of its rarity and various histological types, evidence in support of standard systemic therapies in this patient population remains limited.

Recently, the effectiveness of targeted therapy for specific oncogenic driver alterations in SGC has been established (4–7). Because of the relatively high anti-tumor efficacy and manageable toxicity profile of these agents, the National Comprehensive Cancer Network (NCCN) guidelines state they are a useful therapeutic option for those who harbor the specific alterations (3). However, the majority of patients with SGC do not have these targets, and are accordingly treated with conventional cytotoxic chemotherapy, represented by taxane and platinum as a monotherapy or combination therapy. Of note, docetaxel plus cisplatin and paclitaxel plus carboplatin have been relatively well examined and provided an ORR of 11.5%–54.5% and median overall survival (mOS) of 12–26.5 months in phase II trials and retrospective studies (8–13). Nevertheless, further validation of these conventional therapies is worthwhile, particularly with regard to why treatment efficacy varies among the various histological subtypes. Moreover, no report has compared the efficacy and safety of these two approaches.

Here, we aimed to evaluate the efficacy and safety of taxane and platinum in combination, including docetaxel plus cisplatin and paclitaxel plus carboplatin, in patients with recurrent or metastatic (R/M) SGC. We also performed subgroup analyses by type of regimen and histological subtype to determine whether distinct populations benefit from a specific regimen.

## Materials and methods

### Patient selection

We retrospectively reviewed SGC patients treated with combination chemotherapy with taxane and platinum from January 2000 to September 2021 at the National Cancer Center Hospital East, Japan. The cut-off date was April 1st, 2022. Inclusion criteria were as follows: (1) pathologically proven SGC, (2) not suitable for local therapy, (3) primary site in a major or minor salivary gland, and (4) receipt of at least one course of combination chemotherapy with taxane and platinum in the R/M setting. To extract patients with these conditions, we used a computer-managed search system based on the prescribed regimens, and we then collected their clinical data from each medical record. Patients without target lesions were excluded from the evaluation of antitumor efficacy. This study was approved by the Institutional review Board of the National Cancer Center Hospital East.

### Treatment

The docetaxel plus cisplatin regimen consisted of docetaxel at a dose of 60 mg/m2 plus cisplatin at a dose of 70 mg/m2 on day 1, repeated every 21 days. After completion of six cycles of combination therapy, treatment could continue as maintenance therapy consisting of docetaxel monotherapy at a dose of 60 mg/m2 on day 1, repeated every 21 days. The paclitaxel plus carboplatin regimen consisted of paclitaxel at a dose of 100 mg/m2 plus carboplatin at a dose of area under the plasma concentration-time curve (AUC) = 2.5 on day 1 and 8, repeated every 21 days. Treatment continued until disease progression or the development of intolerable toxicity. If intolerable toxicity to carboplatin appeared, treatment could be continued as maintenance therapy consisting of paclitaxel monotherapy at a dose of 100 mg/m2 on day 1 and 8 every 21 days. The selection of regimen was determined through discussion between the attending physician and the patient themself from the viewpoint of the patient’s organ function, age and performance status, and the patient’s preference in consideration of expected toxicities and administration schedule (docetaxel plus cisplatin is given in an inpatient setting, while paclitaxel plus carboplatin can be administrated in an outpatient setting). In both regimens, dose modification and delay during the treatment schedule were allowed at the physician’s discretion. When combination therapy was discontinued due to toxicity, a switch to maintenance therapy at that time was acceptable in both regimens. Written informed consent for the therapies, including a treatment schedule and expected adverse events, was obtained from each patient. Besides, this study for summarizing their clinical information was approved by the Institutional review Board of the National Cancer Center Hospital East.

### Evaluation of efficacy and statistical analysis

Clinical tumor response to treatment was evaluated radiographically according to primarily Response Evaluation Criteria in Solid Tumors (RECIST) ver. 1.1 using computerized tomography. PFS and OS were calculated by the Kaplan-Meier method and compared using a log-rank test. Hazard ratios were calculated by Cox regression analysis. PFS was calculated from the first day of administration of the taxane and platinum regimen until disease progression or death from any cause. We defined the disease progression of patients with non-target lesions only unequivocal progression containing clinical disease progression. OS was defined as the period from the first admission day of either regimen until death from any cause. Patients who were lost to follow-up were censored at the date of last follow-up. ORR was defined as complete response and partial response rates. Disease control rate (DCR) was defined as complete response, partial response, and stable disease rate. Subgroup analyses by treatment regimen and histological type were performed. Toxicity during the objective treatment period was graded using the Common Toxicity Criteria for Adverse Events (CTCAE version 4.0). All statistical analyses were performed with EZR (version 1.51; Saitama Medical Center, Jichi Medical University, Saitama, Japan), which is a graphical user interface for R (The R Foundation for Statistical Computing, Vienna, Austria; version 4.1.1).

## Results

### Patient characteristics

Forty patients were identified. Their characteristics are summarized in **Table 1**. Median age was 60 years (range, 31–77 years), and ECOG performance status (PS) of 0/1/2 was 21/15/3, respectively. Median baseline of creatinine clearance using the Cockcroft-Gault formula was 85.9 mL/min (range, 43.3-140.2). The most common histological type was adenoid cyst carcinoma (AdCC) (n=10), followed by adenocarcinoma not otherwise specified (ANOS) (n=8), salivary duct carcinoma (SDC) (n=8) and Carcinoma ex pleomorphic adenoma (CEPA) (n=6). The positivity of androgen receptor (AR) and human epidermal growth factor receptor 2 (HER2) in representative histological subtypes were 25.0% and 12.5% in ANOS, 62.5% and 37.5% in SDC, 50.0% and 33.3% in CEPA, respectively (**Table 1**). Furthermore, patient and tumor characteristics according to the histological subtypes (AdCC *vs.* others) and regimens are shown in **Supplementary Table 1**.

**Table 1:** Patient and tumor characteristics.

	N = 40 (%)
Median age, years [range]	60 [31–77]
Gender	
Male	26 (65)
Female	14 (35)
ECOG PS	
0	21 (53)
1	15 (38)
2	3 (8)
Primary site	
Parotid gland	22 (55)
Submandibular gland	12 (30)
Minor salivary gland	6 (15)
Histology	
Mucoepidermoid carcinoma	2 (5)
Adenoid cystic carcinoma	10 (25)
Acinic cell carcinoma	1 (3)
Adenocarcinoma, NOS (ANOS)	8 (20)
Salivary duct carcinoma (SDC)	8 (20)
Carcinoma ex pleomorphic adenoma (CEPA)	6 (15)
Poorly differentiated carcinoma	4 (10)
Carcinoma, NOS	1 (3)
Prior systemic therapy line^†^	
0	29 (72.5)
1	9 (22.5)
2	2 (5)
**Median baseline of creatinine clearance using the Cockcroft-Gault formula** (mL/min) [range]	85.9 [43.3–140.2]
Hormone receptor expression (overall)	
AR-positive and HER2-positive	6 (15)
AR-positive and HER2-negative	5 (13)
Both negative or uncertain	29 (73)
Hormone receptor expression in representative subtypes	
ANOS (n=8)	
AR-positive	4 (25)
HER2-positive	2 (12.5)
SDC (n=8)	
AR-positive	5 (62.5)
HER2-positive	3 (37.5)
CEPA (n=6)	
AR-positive	3 (50.0)
HER2-positive	2 (33.3)
Prior hormone therapy	
Yes	8 (20)
No	32 (80)
Next-generation sequencing	
Yes	15 (38)
No	25 (62)

^†^The number indicates the treatment line in which chemotherapy and hormone therapy were used as systemic therapy for R/M SGC. AR, androgen receptor; HER2, human epidermal growth factor receptor 2.

### Treatment outcome

**Figure 1:**
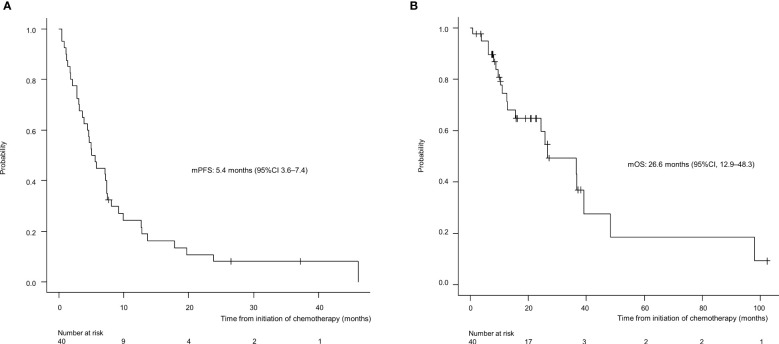
Progression-free survival **(A)** and overall survival **(B)** for the entire population (N=40). mPFS, median progression-free survival; CI, confidence interval.

**Figure 2:**
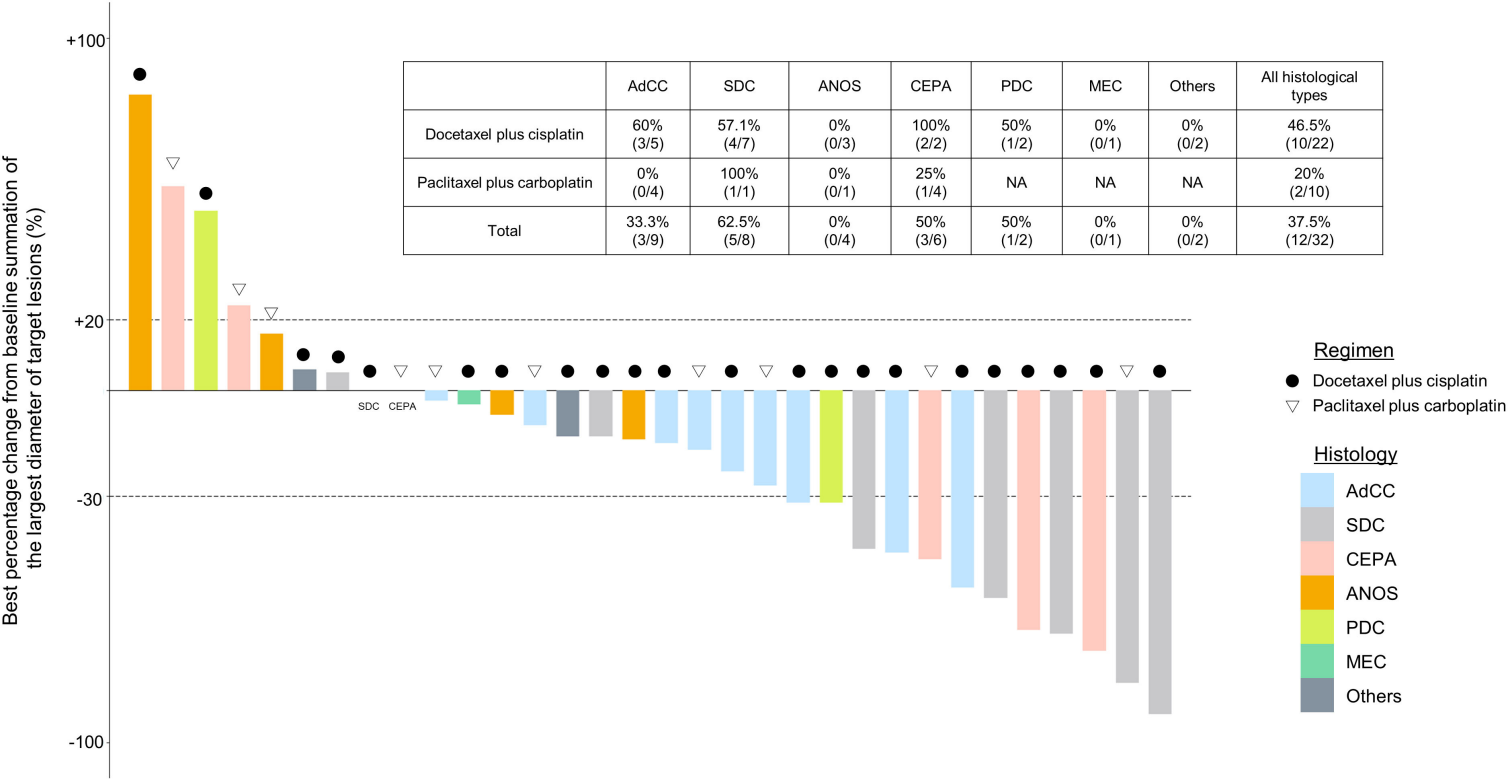
Clinical efficacy analysis with waterfall plots of patients evaluable by RECIST (n=32). NA, not applicable; AdCC, Adenoid cystic carcinoma; SDC, Salivary duct carcinoma; ANOS, Adenocarcinoma, NOS; CEPA, Carcinoma ex pleomorphic adenoma, PDC, Poorly differentiated carcinoma; MEC, Mucoepidermoid carcinoma.

For all 40 patients, median follow-up time was 15.8 months (range, 0.8–102.3 months) at the cut-off date. The mPFS and mOS were 5.4 months (95% CI 3.6-7.4 months) and 26.6 months (95% CI 12.9- 48.3 months) in the total population (**Figure 1**); 4.5 months (95%CI 0.5-17.7) and 30.6 months (95%CI 24.3-NA) in the AdCC group, 5.7 months (95%CI 3.2-7.5) and 26.6 months (11.0-39.0 months) in non-AdCC group, respectively. Thirty-two patients had target lesions evaluable by RECIST, and the ORR and DCR in this population were 37.5% and 87.5% in the total population, 33.3% and 0% in the AdCC group, 39.1% and 17.4% in the non-AdCC group, respectively (**Table 2** and **Supplementary Table 2**). Twenty-three patients (72%) achieved any tumor shrinkage with treatment, with a median change in the sum of tumor diameters from baseline of -18.9% (range, -92-+84%). Regarding treatment regimen and reasons for choosing the regimen, 29 patients were treated with docetaxel plus cisplatin, 11 with paclitaxel plus carboplatin; six of 11 patients in the paclitaxel plus carboplatin group requested the regimen preferring its outpatient-based treatment, and the remaining five had medical complications which hamper using cisplatin, such as cardio-pulmonary dysfunction (n=3), renal impairment (n=1) as well as advanced age (> 75 years old, n=1) (**Supplementary Table 1**). In the docetaxel plus cisplatin group, five patients proceeded to the docetaxel maintenance phase, and two of them terminated the treatment due to disease progression after six docetaxel administrations, and the other two experienced treatment termination due to adverse events after 19 and ten docetaxel administrations each, resulting that one in the group was under treatment with docetaxel monotherapy as of data cut-off(**Supplementary Figure 1**). Median follow-up time was 19.2 months (range, 0.8–102.3 months) for docetaxel plus cisplatin group and 10.3 months (range, 2.1–38.6 months) for the paclitaxel plus carboplatin group. Although the limited subject number and uneven background between the two groups might have impacted the results, analysis to estimate prognosis by type of treatment regimen was attempted. For PFS, docetaxel plus cisplatin showed a statistically significant prolongation of outcome compared with paclitaxel plus carboplatin (mPFS: 7.2 months *vs.* 2.8 months, log-rank p-value; 0.01, hazard ratio [HR]; 0.39 (95% confidence interval [CI], 0.19-0.83) (**Supplementary Figure 2**). Further, a trend toward favorability was also seen in the docetaxel plus cisplatin group (mOS: 36.6 months *vs.* 12.9 months, log-rank p-value; 0.25, HR; 0.54 (95%CI, 0.19-1.56). Antitumor efficacy in the 32 patients who were evaluable by RECIST is shown in **Figure 2** and **Supplementary Table 2**. ORR and DCR by docetaxel plus cisplatin and paclitaxel plus carboplatin were 46.5% *vs.* 20.0% and 90.9%, and 80.0%, respectively (**Supplementary Table 2**). Further detailed assessment with consideration to the impact of histological type on efficacy revealed a distinctive relationship between the two; as one example, docetaxel plus cisplatin showed relatively robust antitumor efficacy in AdCC compared with paclitaxel plus carboplatin (ORR: 60% *vs.* 0%) (**Figure 2**). Moreover, in the subgroup analysis focusing on the AdCC population in this study at least, the docetaxel plus cisplatin group showed statistically significantly prolonged PFS compared with the paclitaxel plus carboplatin group (mPFS: 17.7 months *vs.* 2.8 months, log-rank p-value; 0.0237, HR; 0.10 (95% CI, 0.01-0.89) (**Supplementary Figure 3**).

**Table 2:** Antitumor efficacy in 32 patients who be evaluable by RECIST.

	n = 32^†^ (%)
BOR	
Complete response	1 (3)
Partial response	11 (31)
Stable disease	16 (50)
Progressive disease	4 (13)
**ORR**, %	37.5
**DCR**, %	87.5
Tumor shrinkage by the treatment	
Yes	23 (72)
No	9 (28)
**Mean change in the sum of tumor diameter from baseline**, % [range]	-18.9 [-92 to +84]
**Mean change in the sum of tumor diameter from baseline**, % [range]	-18.9 [-92 to +84]
	n = 32^†^ (%)
BOR	
Complete response	1 (3)
Partial response	11 (31)
Stable disease	16 (50)
Progressive disease	4 (13)
**ORR**, %	37.5
**DCR**, %	87.5
Tumor shrinkage by the treatment	
Yes	23 (72)
No	9 (28)
**Mean change in the sum of tumor diameter from baseline**, % [range]	-18.9 [-92 to +84]

^†^Data were analyzed in 32 evaluable patients. BOR, best overall response; ORR, objective response rate; DCR, disease control rate.

### Safety

Toxicities experienced during treatment are listed in **Table 3**. The most common grade 3/4 adverse event was a decrease in neutrophil count (59% in the docetaxel plus cisplatin group and 27% in the paclitaxel plus carboplatin group). Regarding the use of Granulocyte colony-stimulating factors(G-CSF), primary G-CSF prophylaxis (G-CSF administration in the first cycle of chemotherapy before the onset of neutropenia) was not performed in both groups. While ten (37.9%) patients in the docetaxel plus cisplatin group were administered the agent after the occurrence of neutropenia. Furthermore, the age of these patients who recured G-CSF administration tended to higher than those who did not (average age: 64 *vs.* 55, p=0.08). On the other hand, no patients in the paclitaxel plus carboplatin group were given G-CSF throughout the treatment. A few patients experienced febrile neutropenia (3% in the docetaxel plus cisplatin group and 0% in the paclitaxel plus carboplatin group). No treatment-related death was observed in any patient.

**Table 3:** Adverse event.

	Any grade		Grade 3/4	
	Docetaxel plus cisplatin	Paclitaxel plus carboplatin	Docetaxel plus cisplatin	Paclitaxel plus carboplatin
	n = 29 (%)	n = 11 (%)	n= 29 (%)	n = 11 (%)
**Haematological**				
Neutropenia	17 (59)	5 (45)	17 (59)	3 (27)
Febrile neutropenia	1 (3)	0 (0)	1 (3)	0 (0)
Platelet count decreased	1 (3)	0 (0)	1 (3)	0 (0)
**Non-haematological**				
Malaise	9 (31)	3 (27)	1 (3)	0 (0)
Nausea	10 (34)	5 (45)	0 (0)	0 (0)
Alopecia	4 (14)	3 (27)	0 (0)	0 (0)
Creatinine increased	4 (14)	0 (0)	0 (0)	0 (0)
Diarrhea	4 (14)	2 (18)	0 (0)	0 (0)
Constipation	3 (10)	2 (18)	0 (0)	0 (0)
Dysgeusia	3 (10)	2 (18)	0 (0)	0 (0)
AST increased	3 (10)	0 (0)	0 (0)	0 (0)
ALT increased	3 (10)	0 (0)	1 (3)	0 (0)
Edema	3 (10)	0 (0)	0 (0)	0 (0)
Hyperglycemia	3 (10)	0 (0)	2 (7)	0 (0)
Peripheral sensory neuropathy	2 (7)	5 (45)	0 (0)	0 (0)
Hypomagnesemia	2 (7)	0 (0)	0 (0)	0 (0)
PPE	1 (3)	2 (18)	0 (0)	0 (0)
Hypokalemia	1 (3)	0 (0)	0 (0)	0 (0)
Infusion related reaction	0 (0)	1 (9)	0 (0)	0 (0)

All events were graded according to common toxicity criteria for adverse events version 4.0. AST, aspartate aminotransferase; ALT, alanine aminotransferase; PPE, palmar-plantar erythrodysesthesia.

## Discussion

In this study, we comprehensively evaluated the efficacy and safety of two widely used combination chemotherapies based on taxane and platinum in patients with SGC. Furthermore, in a subgroup analysis, we revealed for the first time that efficacy might differ according to histological subtype; notably, the combination of paclitaxel plus carboplatin showed unfavorable antitumor efficacy and prognosis compared with docetaxel plus cisplatin in patients with AdCC (ORR: 0% *vs.* 60.0%, mPFS: 2.8 months *vs.* 17.7 months, mOS: 24.2 months *vs.* 42.4 months).

Treatment for R/M SGC generally consists of systemic therapy, as with other cancer types. Among therapies, recent progress in precision medicine has led to molecular-targeted therapy for subjects harboring the corresponding therapeutically targetable alteration. However, this treatment is suitable for only a small fraction of the population; moreover, systems for evaluating these alterations have yet to be generalized and widely distributed. For instance, the Center for Cancer Genomics and Advanced Therapeutics reported that only 7.8% of all Japanese cancer patients tested for comprehensive genomic profiling underwent drug treatment based on genomic alterations (4). Thus, R/M SGC patients who do not have these targets or the opportunity to receive a companion diagnosis are still treated with conventional cytotoxic chemotherapy. Nevertheless, no standard regimen for these patients has yet been established, as confirmed by the NCCN guidelines, which also state that no preferred regimen exists. Indirect comparisons suggest that combination therapy, represented by taxane and platinum, is more effective in terms of response rate and progression-free survival than monotherapy (5–7, 14–17) (**Table 4**). Our present results appear to mirror these recent findings, with efficacy of combination therapy in various histological types showing ORRs ranging from 39%-54.5%, PFS of 6.5-8.4 months, and mOS of 18.8-26.5 months (14, 15, 17). The adverse events of each regimen were tolerable. The most frequent grade 3/4 adverse event was neutropenia (59% with docetaxel plus cisplatin and 27% with paclitaxel plus carboplatin); however, febrile neutropenia occurred in only one case in the docetaxel plus cisplatin group. In contrast, grade 3/4 neutropenia was more common in previous reports, for example at 95% with docetaxel plus cisplatin and 53% with paclitaxel plus carboplatin (14, 17). The reason for these contrasting findings may be the dose difference, as shown in **Table 4**; generally, the dose per unit time in our regimen was relatively lower than in the other studies. Indeed, the optimal dose of combination chemotherapy with taxane and platinum for SGC patients remains unknown. Nevertheless, our regimens appear to represent a well-balanced therapeutic option in terms of both efficacy and safety.

**Table 4:** The summary of literature reports on taxane and/or platinum regimen for salivary gland carcinoma.

Author	Year	Phase	Regimen^†^	N	mPFS(mo)	mOS (mo)	ORR (%)							
							All	AdCC	SDC	ANOS	CEPA	PDC	MEC	Others
Licitra et al.	1991	II	Cisplatin (100mg/m^2^, d1)	25	7	14	16 (4/25)	15 (2/13)	NA	0 (0/5)	NA	NA	20 (1/5)	50 (1/2)
Airoldi et al.	2000	II	Paclitaxel (175mg/m^2^, d1) Carboplatin (AUC5.5, d1)	14	NA	12.5	14 (2/14)	20 (2/10)	NA	0 (0/1)	NA	0% (0/2)	0 (0/1)	NA
Gilbert et al.	2006	II	Paclitaxel (200mg/m^2^, d1)	45	4	12.5	18 (8/45)	0 (0/14)	NA	29 (5/17)	NA	NA	21 (3/14)	NA
Nakano et al.	2016	retro	Paclitaxel (200mg/m^2,^ d1) Carboplatin (AUC6, d1)	38	6.5	26.5	39 (15/38)	11 (1/9)	39 (7/18)	64 (7/11)				
										4	4	NA	1	2
Okada et al.	2019	retro	Docetaxel (70mg/m^2^, d1) Carboplatin (AUC5. d1)	24	8.4	26.4	42 (10/24)	0 (0/1)	50 (6/12)	50 (2/4)	NA	NA	0 (0/1)	33 (2/6)
Fukuda et al.	2021	retro	Paclitaxel (200mg/m^2^, d1) Carboplatin (AUC6, d1)	26	8.1	22.3	11.5 (3/26)	11.5 (3/26)	NA	NA	NA	NA	NA	NA
Imamura et al.	2021	II	Docetaxel (75mg/m2, d1) Cisplatin (75mg/m^2^, d1)	11	6.6	18.8	54.5	50 (2/4)	67 (2/3)	67 (2/3)	NA	NA	NA	0 (0/1)
Current study.	2023	retro	Docetaxel (60mg/m^2^, d1) Cisplatin (70mg/m^2^, d1)	22	7.2	36.6	46.5 (10/22)	60 (3/5)	57.1 (4/7)	0 (0/3)	100 (2/2)	50 (1/2)	0 (0/1)	0 (0/2)
			Paclitaxel (100mg/m^2^, d1,8) Carboplatin (AUC2.5, d1,8)	10	2.8	12.9	20 (2/10)	0 (0/4)	100 (1/1)	0 (0/1)	25 (1/4)	NA	NA	NA

^†^All regimens given over three weeks. ORR, objective response rate; mPFS, median progression-free survival; mOS, median overall survival; AdCC, adenoid cystic carcinoma; SDC, salivary duct carcinoma; ANOS, adenocarcinoma, not otherwise specified; CEPA, carcinoma ex pleomorphic adenoma, PDC, poorly differentiated carcinoma; MEC, mucoepidermoid carcinoma; NA, not applicable.

AdCC is characteristically slow-growing but has a high recurrence rate and is considered to draw a line from other SGC subtypes, at least regarding its treatment strategy; however, evidence on systemic therapy for the disease is not well established. The American Society of Clinical Oncology guidelines and NCCN guidelines recommend lenvatinib, a multi-targeted tyrosine kinase inhibitor, for AdCC (category 2B treatment in the NCCN guidelines), based on the results of phase II trials in a relatively small number of patients (8–10). Antitumor efficacy is modest, however, with an ORR of 10.5-15.6%, and worldwide adoption as the standard of care has not been achieved. Moreover, the disease rarely harbors therapeutically targetable alterations (11). Against this background, exploration of treatment options has continued, including the reevaluation of conventional cytotoxic chemotherapy. Notably, although taxane and platinum as monotherapy has been recognized to provide limited anti-tumor efficacy, as shown in **Table 4** (5, 7), the recent phase II trial mentioned above reported that docetaxel plus cisplatin provided an ORR of 54.5% in 11 SGC patients, and that 50% (2/4) of AdCC patients achieved a partial response (12, 17), as similarly seen in our present study (60%, 3/5). In contrast, we found for the first time that paclitaxel plus carboplatin might lead to unfavorable treatment outcome in this population (ORR: 0%, mPFS: 2.8 months). These results, although not conclusive due to the limited patient number and inability to determine the difference in efficacy, may suggest that for AdCC patients who require systemic therapy and are able to tolerate docetaxel plus cisplatin, this regimen is the preferred option, particularly given the encouraging efficacy over that in previous reports of AdCC (ORRs: 15.6-43%) (9, 13, 18, 19).

This study has several limitations. First, the subgroup analysis on the potential impact of the type of regimen (i.e., docetaxel plus cisplatin *vs.* paclitaxel plus carboplatin) on efficacy was hampered by the heterogeneous patient characteristics, including the unbalanced number of enrolled patients between the two, and the lack of clarity in regimen selection due to selection bias from the retrospective study design. A further randomized trial would therefore provide a more conclusive answer for this clinically significant issue. Second, unfortunately, the standardized treatment schedule and dose of the taxane and platinum have yet to be established worldwide, and we also could not reach a conclusive perspective on it, especially in the combination of paclitaxel plus carboplatin, and verification of the meaning of switching to the maintenance of docetaxel in the docetaxel plus cisplatin, through the current work. Third, despite the significance of examining therapeutically targetable alterations in the SGC population, 62% of our present patients were not examined, as the cohort includes the subjects treated before comprehensive genome profiling was covered by insurance in 2019. The remaining patients (38%) had no targetable alterations, at the time at least; however, we should note that this fact might cause a biased result that does not match the current clinical situation and that further study may reveal the true efficacy of these combinations in subjects who do not have such targets, as well as identify predictive markers in patients who would substantially benefit from these regimens.

## Conclusion

The combination of taxane and platinum is a chemotherapeutic option for patients with salivary gland carcinoma. In contrast, paclitaxel plus carboplatin may be less effective in certain situations, such as in patients with AdCC.

## Data availability statement

The original contributions presented in the study are included in the article/**Supplementary Material**. Further inquiries can be directed to the corresponding author.

## Ethics statement

The studies involving human participants were reviewed and approved by Institutional Review Board of the National Cancer Center Hospital East. Written informed consent for participation was not required for this study in accordance with the national legislation and the institutional requirements.

## Author contributions

RO and KI participated in formulating the study concept and design, data curation, data interpretation, and drafting of the manuscript. TE participated in data interpretation and drafting of the manuscript. MT supervised the study and revised the manuscript. All authors provided critical revisions and approved the final manuscript. All authors contributed to the article.
