# Supplementary material for: Combination chemotherapy with taxane and platinum in patients with salivary gland carcinoma: a retrospective study of docetaxel plus cisplatin and paclitaxel plus carboplatin
Source: Front Oncol. 2023 Jun 15;13:1185198. doi: 10.3389/fonc.2023.1185198 (PMC10311248; doi:10.3389/fonc.2023.1185198)
Supplement: Supplementary file 1 [file DataSheet_1.docx]

# Supplementary Figures and Tables

**Supplementary Figure 1.** Patient flow diagram of treatment delivery.


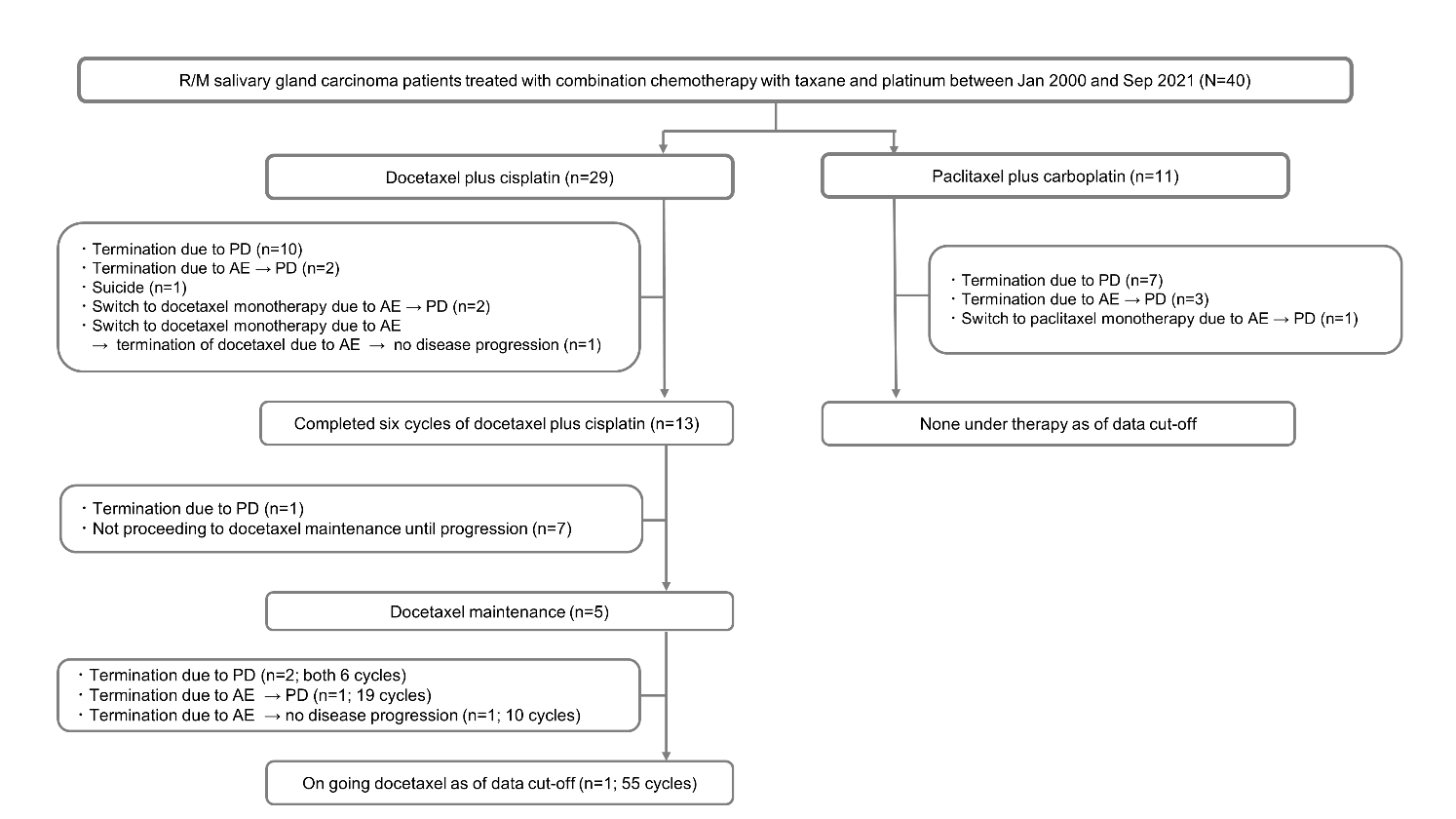


## Abbreviations: R/M, recurrent or metastatic; PD, progressive disease; AE, adverse event.

## Supplementary Figure 2. Progression-free survival (A) and overall survival (B) by treatment regimen (N=40).


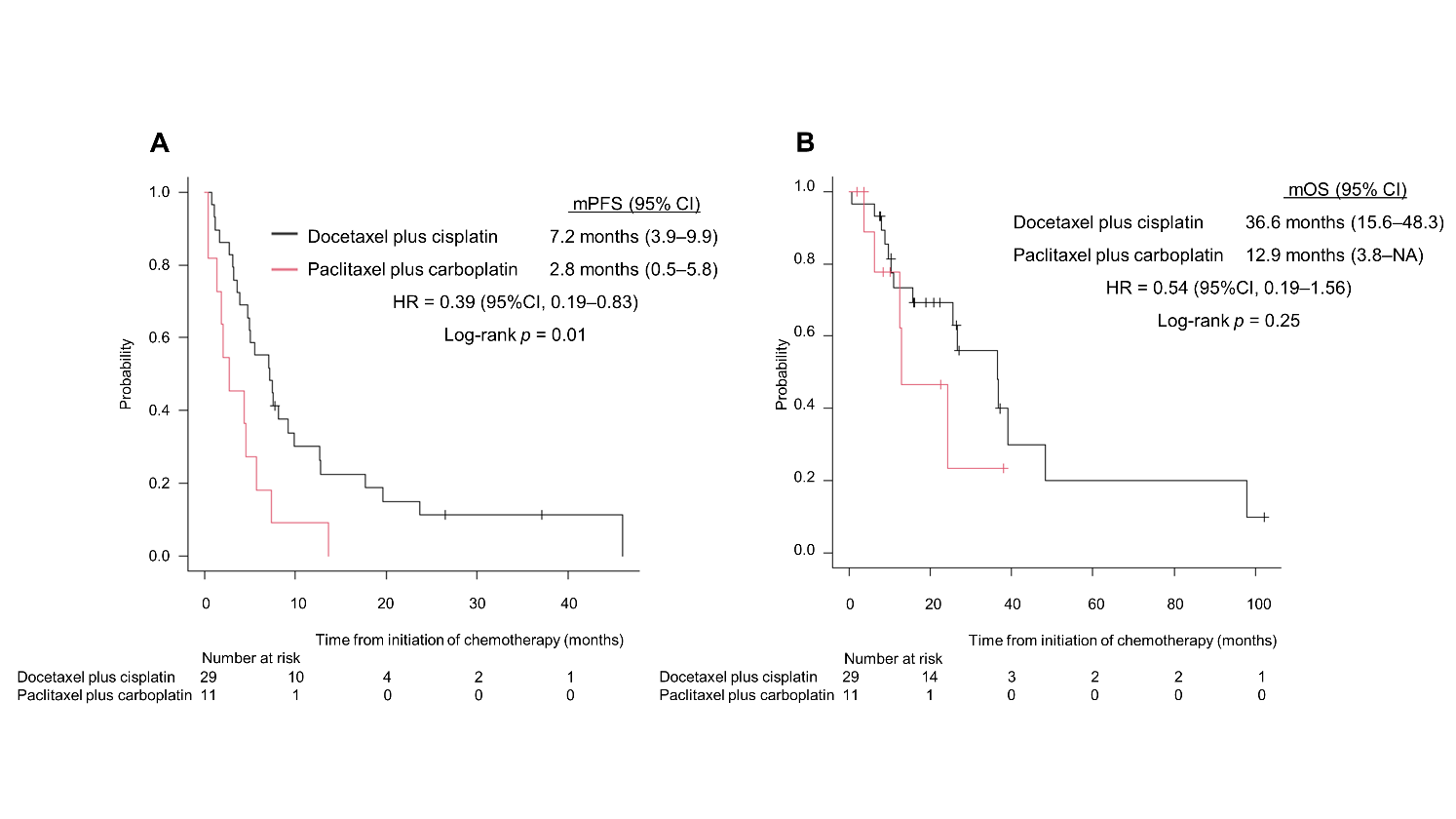


## Abbreviations: mPFS, median progression-free survival; CI, confidence interval; HR, hazard ratio.

## Supplementary Figure 3. Progression-free survival (A) and overall survival (B) by treatment regimen in patients with adenoid cystic carcinoma (n=10).


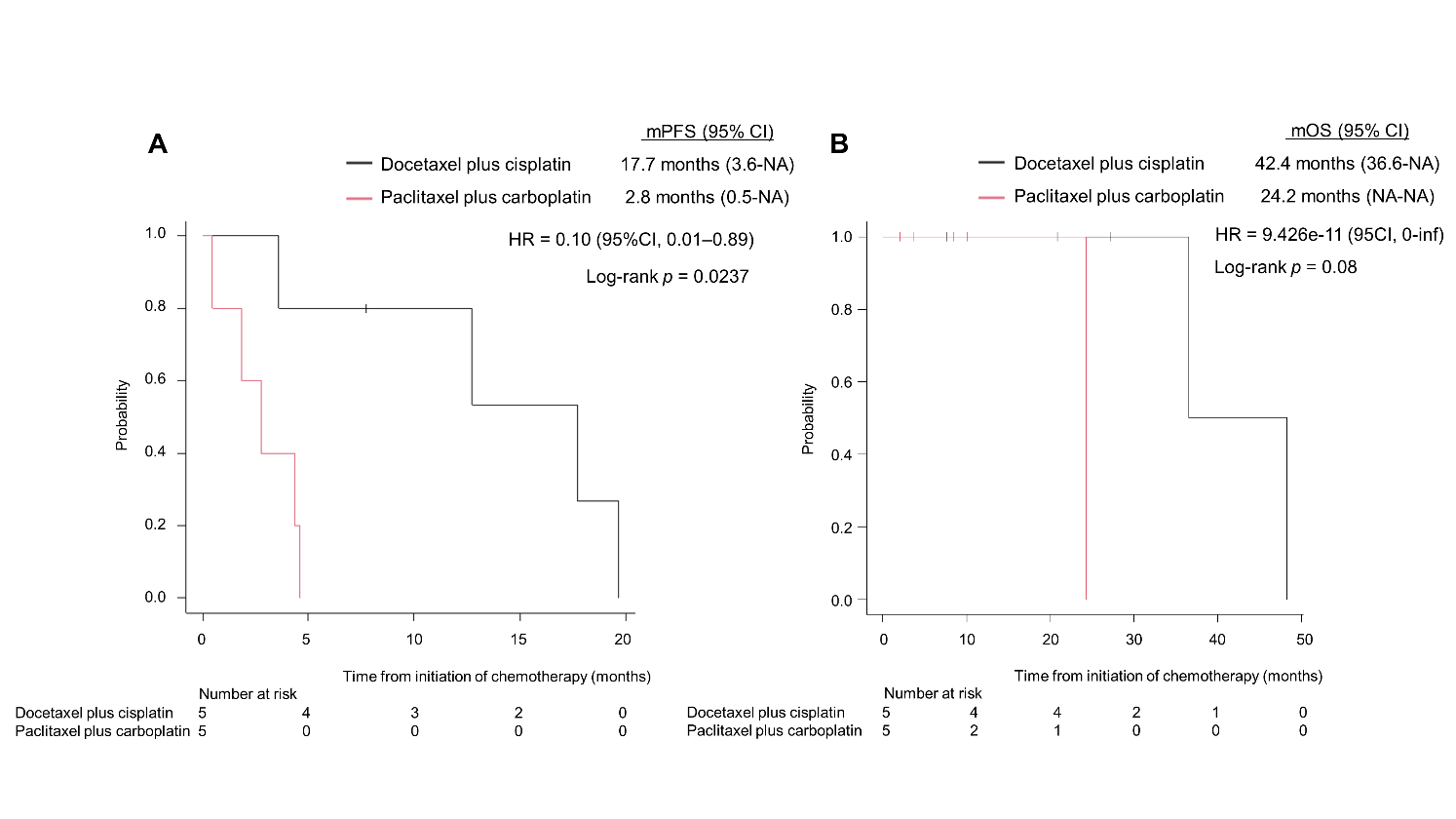


Abbreviations: mPFS, median progression-free survival; CI, confidence interval; HR, hazard ratio.

**Supplementary Table 1.** Patient and tumor characteristics according to histological subtypes (AdCC vs. others) and treatment regimens

|  | **Adenoid cystic carcinoma**  n = 10 | | **Other histological types**  n = 30 | |
| --- | --- | --- | --- | --- |
|  | **Docetaxel plus cisplatin**  n = 5 (%) | **Paclitaxel plus carboplatin**  n = 5 (%) | **Docetaxel plus cisplatin**  n = 24 (%) | **Paclitaxel plus carboplatin**  n = 6 (%) |
| **Median age**, years [range] | 53 [31–62] | 58 [46–73] | 63 [38–75] | 61 [40–77] |
| **Gender**  Male  Female | 4 (80)  1 (20) | 1 (45)  4 (55) | 17 (71)  7 (29) | 4 (67)  2 (33) |
| **ECOG PS**  0/1/2 | 4 / 1 / 0  (80 / 20 / 0) | 1 / 2 / 2  (20 / 40 / 40) | 13 / 10 / 1  (54 / 42 / 4) | 3 / 2 / 1  (50 / 33 / 17) |
| **Primary site**  Parotid gland  Submandibular gland  Minor salivary gland | 2 (40)  1 (20)  2 (40) | 4 (80)  0 (0)  1 (20) | 13 (54)  9 (38)  2 (8) | 3 (50)  2 (33)  1 (17) |
| **Histology**  Mucoepidermoid carcinoma  Adenoid cystic carcinoma  Acinic cell carcinoma  Adenocarcinoma, NOS  Salivary duct carcinoma  Carcinoma ex pleomorphic adenoma  Poorly differentiated carcinoma  Carcinoma, NOS | -  5 (100) -  -  -  -  -  - | -  5 (100)  -  -  -  -  -  - | 2 (8)  -  1 (4)  7 (29)  7 (29)  2 (8)  4 (16)  1 (4) | 0 (0)  -  0 (0)  1 (17)  1 (17)  4 (67)  0 (0)  0 (0) |
| **Prior systemic therapy line**^†^  0  1  2 | 5 (100)  0 (0)  0 (0) | 5 (100)  0 (0)  0 (0) | 17 (71)  5 (21)  2 (8) | 2 (33)  4 (66)  0 (0) |
| **Median baseline of creatinine clearance using the Cockcroft-Gault formula** (mL/min) [range] | 105.7  [57.8 to 121.3] | 109.0  [56.5 to 136.0] | 81.4  [59.4 to 140.2] | 89.2  [43.3 to 114.8] |
| **Hormone receptor expression**  AR-positive and HER2-positive  AR-positive and HER2-negative  Both negative or uncertain | 0 (0)  0 (0)  5 (100) | 0 (0)  0 (0)  5 (100) | 3 (13)  4 (16)  17 (71) | 3 (50)  1 (17)  2 (33) |
| **Prior hormone therapy**  Yes  No | 0 (0)  5 (100) | 0 (0)  5 (100) | 6 (24)  18 (76) | 2 (33)  4 (67) |
| **Next‑generation sequencing**  Yes  No | 2 (40)  3 (60) | 3 (60)  2(40) | 6 (24)  18 (76) | 4 (67)  2 (33) |
| **Reason for choosing paclitaxel plus**  **carboplatin, not docetaxel plus cisplatin** Patient’s preference  Cisplatin-ineligible  　 Cardio-pulmonary dysfunction  Renal impairment  Advanced age (> 75 years old) | - | 4 (80)  1 (20)  1 (20)  0 (9)  0 (9) | - | 2 (33)  4 (67)  2 (33)  1 (17)  1 (17) |

^†^ The number indicates the treatment line in which chemotherapy and hormone therapy were used as systemic therapy for R/M SGC. Abbreviations: AdCC, adenoid cystic carcinoma; AR, androgen receptor; HER2, human epidermal growth factor receptor 2.

**Supplementary Table 2.** Antitumor efficacy according to histological subtypes (AdCC vs. others) and treatment regimens

|  | **Adenoid cystic carcinoma**  n = 9^†^ | | **Other histological types**  n = 23^†^ | |
| --- | --- | --- | --- | --- |
|  | **Docetaxel plus cisplatin**  n = 5^†^ (%) | **Paclitaxel plus carboplatin**  n = 4^†^ (%) | **Docetaxel plus cisplatin**  n = 17^†^ (%) | **Paclitaxel plus carboplatin**  n = 6^†^ (%) |
| **BOR**  Complete response  Partial response  Stable disease  Progressive disease | 0 (0)  3 (60)  2 (40)  0 (0) | 0 (0)  0 (0)  4 (100)  0 (0) | 1 (6)  6 (35)  8 (47)  2 (12) | 0 (0)  2 (33)  2 (33)  2 (33) |
| **ORR**, % | 60.0 | 0 | 41.1 | 33.3 |
| **DCR**, % | 100.0 | 100.0 | 88.2 | 66.6 |
| **Tumor shrinkage by treatment**  Yes  No | 5 (100)  0 (0) | 4 (100)  0 (0) | 12 (71)  5 (29) | 2 (33)  4 (66) |
| **Mean change in the sum of tumor diameter from baseline**, % [range] | -34.6  [-56 to -15] | -14.3  [-27 to -3] | -20.2  [-92 to +84] | -5.5  [-83 to +58] |

^†^Data were analyzed in 32 evaluable patients. Abbreviations: AdCC, adenoid cystic carcinoma; BOR, best overall response; ORR, objective response rate; DCR: disease control rate.
